# Separate-channel analysis of two-channel microarrays: recovering inter-spot information

**DOI:** 10.1186/1471-2105-14-165

**Published:** 2013-05-26

**Authors:** Gordon K Smyth, Naomi S Altman

**Affiliations:** 1 Walter and Eliza Hall Institute of Medical Research, Melbourne, Vic 3052, Australia; 2 Department of Mathematics and Statistics, University of Melbourne, Vic 3010, Australia; 3 Department of Statistics, The Pennsylvania State University, University Park, PA 16802–2111, USA

**Keywords:** Loop design, Unconnected design, Reference design, Intraclass correlation, False discovery rate, Power, Efficiency

## Abstract

**Background:**

Two-channel (or two-color) microarrays are cost-effective platforms for comparative analysis of gene expression. They are traditionally analysed in terms of the log-ratios (M-values) of the two channel intensities at each spot, but this analysis does not use all the information available in the separate channel observations. Mixed models have been proposed to analyse intensities from the two channels as separate observations, but such models can be complex to use and the gain in efficiency over the log-ratio analysis is difficult to quantify. Mixed models yield test statistics for the null distributions can be specified only approximately, and some approaches do not borrow strength between genes.

**Results:**

This article reformulates the mixed model to clarify the relationship with the traditional log-ratio analysis, to facilitate information borrowing between genes, and to obtain an exact distributional theory for the resulting test statistics. The mixed model is transformed to operate on the M-values and A-values (average log-expression for each spot) instead of on the log-expression values. The log-ratio analysis is shown to ignore information contained in the A-values. The relative efficiency of the log-ratio analysis is shown to depend on the size of the intraspot correlation. A new separate channel analysis method is proposed that assumes a constant intra-spot correlation coefficient across all genes. This approach permits the mixed model to be transformed into an ordinary linear model, allowing the data analysis to use a well-understood empirical Bayes analysis pipeline for linear modeling of microarray data. This yields statistically powerful test statistics that have an exact distributional theory. The log-ratio, mixed model and common correlation methods are compared using three case studies. The results show that separate channel analyses that borrow strength between genes are more powerful than log-ratio analyses. The common correlation analysis is the most powerful of all.

**Conclusions:**

The common correlation method proposed in this article for separate-channel analysis of two-channel microarray data is no more difficult to apply in practice than the traditional log-ratio analysis. It provides an intuitive and powerful means to conduct analyses and make comparisons that might otherwise not be possible.

## Background

Microarrays have been the most popular technology for genome-wide profiling of gene expression for the past 15 years. The earliest microarrays used two channels, with two RNA samples separately labeled and competitively hybridized to the same array, as a means of controlling inter-probe variability [1]. Despite the rise of one channel microarrays and other expression profiling technologies, two-channel arrays, also known as two-color arrays, continue to be a cost-effective platform for assessing relative gene expression. The use of two channels is more efficient than one channel for many comparative experiments [2,3]. Microarrays constructed from EST libraries may also be most effective when used with two channels. For species with few genomic resources a common strategy for differential expression studies is to use EST libraries or high throughput sequencing methods to obtain partial transcriptome information and then use the resulting transcripts to develop microarray probes. For example, [4] developed a Nimblegen microarray to investigate disease resistance in apple and [5] used a custom microarray to assess differential gene expression in diseases of a marine flatfish. Bay LK, 2009 [6] used a custom spotted cDNA array to assess differential expression between populations of a reef-building coral.

The traditional and most common approach to the analysis of two-channel gene expression microarrays is to summarize the intensity values in terms of the log-ratios of the two channel intensities for each probe on each array [7]. Most papers on the statistical analysis of two-channel microarrays have also taken this general approach [8,9]. Expression levels measured by neighboring spots on the same array have been shown to be highly correlated [10]. Observations of the two channels from the same physical spot are expected to be even more highly correlated. The practice of analyzing log-ratios in effect takes advantage of this correlation, as the variance of the log-ratio should be smaller than the sum of the variances of two individual positively correlated log-intensity values.

It has been argued however that log-ratio analyses are not fully efficient in that they do not use all the information available in the data [11-13]. A number of papers have popularized the idea of analyzing the individual channel intensities as separate observations [14-17]. One approach is to an estimate a random effect for each microarray spot to account for the correlation between the two channels [16,17]. This approach can be implemented by fitting a mixed model, a linear model with both fixed and random effects, to the expression data for each gene [16,17]. Separate channel analysis has been used to analyze experiments for which two channels were available for some arrays and only one channel for others [18]. Separate-channel approaches to the normalization of two-channel microarray data have also been discussed [19].

Apart from other efficiency gains, separate channel analysis gives the possibility of comparing treatment conditions that are not connected in a two-color experimental design [15]. A two-channel microarray design is said to be connected if every pairwise comparison among treatments can be expressed as a difference of log-ratios. For example, reference designs and loop designs are connected [14]. Unconnected designs contain islands of arrays with treatments that are unlinked by hybridization to the same arrays. For example, if an experiment includes 4 treatments B, C, D and E with treatments B and C always hybridized together and treatments D and E always hybridized together, there is no way to compare treatments B and D using the log-ratio approach. Separate channel models are necessary to analyze such unconnected designs.

A number of papers have shown that the two channel intensities from each spot are usefully represented in terms of the log-ratio (*M*-value) and the average log-intensity over the two channels (*A*-value) for each spot [20,21]. This article reinterprets the *M* and *A*-values in terms of within and between spot contrasts. The usefulness of this partition is shown to arise in good part from the fact that the *M* and *A*-values for a given spot are statistically independent even though the individual log-intensities are highly correlated.

This article goes on to reformulate the mixed model approach in terms of the *M* and *A*-values. This approach not only presents an efficient algorithm for estimating the mixed model but also elucidates the difference between the traditional log-ratio based approach and the analysis of separate-channels. Use of the *M*-values alone for the analysis is shown to discard the between-spot information. The separate-channel approach amounts to recovering information from the between-spot error stratum, i.e., from comparisons among the *A*-values. The efficiency gains of separate-channel analysis are quantified in terms of the intra-spot correlation.

The idea of regularized statistical methods that borrow strength between genomic features is an important recurring theme in genomic data analysis. Statistical research has shown that, across a wide range of multi-parameter problems, improvements in parameter estimation can be made by combining information across the datasets used to estimate the individual parameters [22,23]. This principle is especially important for microarray data analysis with tens of thousands of probe-wise variances to be estimated, and the idea has led to a number of popular regularized microarray methods [24-26]. Regularization of variance components has also been shown to be beneficial for mixed model analyses of microarray data [17]. The article introduces a simple but effective regularization for the spotwise random effects by forcing the intra-spot correlation to be a constant value across all genes in the study. The genewise variances are subsequently regularized using a conjugate empirical Bayes procedure [26]. The treatment of the intra-spot correlation as a global parameter leads to a number of important advantages. The global estimator is very precise, so the intra-spot correlation can be treated as a known parameter at the individual gene level. The permits the mixed model to be transformed into a form suitable for entry into the well-established empirical Bayes analysis pipeline of the limma package [26]. Unlike previous mixed model approaches, this approach leads to test statistics with exact parametric distributions under the null hypothesis, even for experiments with small numbers of samples [26].

The method proposed in this article for separate-channel analysis is no more difficult to apply in practice than the traditional log-ratio analysis and provides an intuitive and powerful means to make comparisons that might otherwise not be possible. The separate channel tests of differential expression are shown to be more statistically powerful than those from the log-ratio approach, leading to reductions in both false discovery and false nondiscovery rates. This article includes several cases studies, which demonstrate the advantages of the separate channel approach and the performance of the proposed regularization approach for the intra-spot correlation and the genewise variances.

## Results and discussion

### Intra-spot correlation

Suppose that a gene expression experiment has been conducted in which 2*n* RNA samples have been hybridized to *n* two-channel microarrays, each printed with the same set of *P* probes. In general, there may be more than one spot on each microarray for the same probe DNA sequence [27], but we will suppose here that all spots are to be treated as if they correspond to independent probes. Note we use “probe” here to refer to the cDNA sequence used in the array design and “spot” to refer to the physical feature printed onto each array. Two-color microarrays yield two intensities values for each spot, one for each channel. Following usual practice, we will call the shorter wave-length channel “green” and the other “red”. Most image analysis softwares yield a foreground and a background intensity for each channel for each spot. We will assume that the foreground intensities have been background corrected, normalized and log-transformed to yield log2 intensities. Write *y*_*gi*1_ and *y*_*gi*2_ for the green and red channel log-intensities, respectively, for probe *g* on array *i*. We will assume that there are no missing values, that is, a finite *y*_*gic*_ is available for all *gic*. In particular, all the background-corrected intensities are assumed to be positive so that the log-intensities are properly defined, as can be ensured by a variety of model-based background correction strategies [28]. The log-intensities are assumed to be normally distributed,

(1) $$y_{\text{gic}}∼N(\mu_{\text{gic}},\sigma_{g}^{2})$$

where *μ*_*gic*_ is an unknown mean that depends on the probe and the treatment conditions applied to channel *c* on array *i*. The unknown $\sigma_{g}^{2}$ is probe-specific but common across arrays for each probe.

We can view each physical spot on each array as a block giving rise to two observations, one for each channel. While it is reasonable to treat log-intensities observed on different arrays as independent, two observations from the same physical feature of the same array must almost inevitably be highly correlated. Hence we assume *y*_*gic*_ and $y_{g^{\prime }i^{\prime }c^{\prime }}$ are independent if $i\neq i^{\prime }$ but that

$$\text{corr}\left(y_{\text{gi}1},y_{\text{gi}2}\right)=\rho_{g}$$

where *ρ*_*g*_ is the unknown intra-spot correlation. We expect *ρ*_*g*_ to be positive to reflect the fact that the two channel observations share any characteristics local to that spot on that array [19].

### The separate channel linear model

Probewise linear models provide a flexible and powerful approach to microarray data analysis [12,16]. We assume that the true mean log-expression values *μ*_*gic*_ can be represented by a linear model

$$\mu_{\text{gic}}=x_{\text{ic}}^{T}\beta_{g}$$

where the design vector *x*_*ic*_ indicates which treatment conditions are applied to the RNA sample hybridized to channel *c* of array *i*, and *β*_*g*_ is an unknown vector of coefficients representing population mean log-intensities or log-fold-changes between the treatment conditions. Often *x*_*ic*_ is a vector of zeros and ones. In matrix notation, the linear model may be written

$$E\left(y_{g}\right)=X\beta_{g}$$

where *y*_*g*_ is the 2*n*-vector of *y*_*gic*_ values for probe *g* and *X* is the design matrix with rows *x*_*ic*_.

The covariance matrix *Var*(*y*_*g*_) is block diagonal, with diagonal elements equal to $\sigma_{g}^{2}$ and off-diagonal elements either zero or equal to $\rho_{g}\sigma_{g}^{2}$ for pairs of observations from the same spot. If an estimate of *ρ*_*g*_ is available, then the linear model coefficient vector *β*_*g*_ can be estimated from the data by generalized least squares,

$${\hat{\beta }}_{g}={\left(X^{T}C_{g}^{-1}X\right)}^{-1}X^{T}C_{g}^{-1}y_{g}$$

where *C*_*g*_ is the correlation matrix obtained by dividing the variances out of Var(*y*_*g*_).

### Estimating the intra-spot correlation by mixed models

One approach to the separate channel linear model is to treat it as a mixed model with each spot a randomized block of size two [12,16]. The mixed linear model can be represented as

$$y_{\text{gic}}=\mu_{\text{gic}}+b_{\text{gi}}+\varepsilon_{\text{gic}}$$

where *b*_*gi*_ is the spot effect for probe *g* on array *i* and *ε*_*gic*_ is a residual error term. The spot and residual effects are both random, with $b_{\text{gi}}∼N(0,\sigma_{\text{bg}}^{2})$ and $\varepsilon_{\text{gic}}∼N(0,\sigma_{\text{eg}}^{2})$. Here $\sigma_{\text{bg}}^{2}$ is the variance component for spot-level variation or, equivalently, for array to array variation. The marginal variance for *y*_*gic*_ is

$$\sigma_{g}^{2}=\sigma_{\text{gb}}^{2}+\sigma_{\text{ge}}^{2}$$

and the intra-spot correlation is

$$\rho_{g}=\frac{\sigma_{\text{gb}}^{2}}{\sigma_{\text{gb}}^{2}+\sigma_{\text{ge}}^{2}}.$$

The mixed linear model obviously assumes that *ρ*_*g*_≥0, because $\sigma_{\text{gb}}^{2}$ cannot be negative. A typical mixed model analysis will obtain REML estimators [29] for $\sigma_{\text{bg}}^{2}$ and $\sigma_{\text{eg}}^{2}$, leading to an estimate for *ρ*_*g*_ as above, and hence to a generalized least squares estimator for *β*_*g*_. Obviously the mixed model constrains the intra-spot correlation to be positive.

Fitting mixed models of this form has been used as the basis for two-channel microarray data analysis, although the test statistics have complex distributions that cannot be derived exactly [16,17]. In this article, we will reformulate the mixed model in order to obtain an exact distributional theory for empirical Bayes test statistics, and in order to clarify the relationship between the mixed model and the traditional log-ratio analysis of microarray data.

### Spot-wise means and differences

Following [20,21], write *M*_*gi*_=*y*_*gi*2_−*y*_*gi*1_ for the log-ratio of red to green intensity for spot *gi* and *A*_*gi*_=(*y*_*gi*2_+*y*_*gi*1_)/2 for the average log-intensity for the spot. These quantities were originally proposed for their use in normalization and quality assurance graphics, with “M” and “A” as mnemonics for Minus and Add respectively. Here we make use of these same quantities as part of a formal statistical analysis.

A key observation is that *M*_*gi*_ and *A*_*gi*_ are uncorrelated. They represent independent contrasts of the correlated observations *y*_*gi*2_ and *y*_*gi*1_. In terms of the above mixed model, *M*_*gi*_ can be interpreted as a within-spot contrast, whereas any comparison of the *A*-values represents a between-spot contrast. The variances are given by

$$\text{var}\left(M_{\text{gi}}\right)=\sigma_{\text{Mg}}^{2}=2\sigma_{g}^{2}\left(1-\rho_{g}\right)$$

and

$$\text{var}\left(A_{\text{gi}}\right)=\sigma_{\text{Ag}}^{2}=\sigma_{g}^{2}\left(1+\rho_{g}\right)/2.$$

We see that the variance of *M* decreases with the intra-spot correlation whereas that of *A* increases. Note in fact that

$$\frac{1}{2}\log\frac{4\sigma_{\text{Ag}}^{2}}{\sigma_{\text{Mg}}^{2}}=\tanh\left(\rho_{g}\right),$$

so that *ρ*_*g*_ can be estimated from the ratio of the two variances.

### Estimating the intra-spot correlation from the *M*-value and *A*-values

We now transform the mixed model for *y* into a model for the *M*-values and *A*-values. This has the effect of transforming the mixed model, in which the observations are not independent but all have the same variance, into a model in which all the observations are independent but the variances are unequal.

Write *M*_*g*_=(*M*_*g*1_,…,*M*_*gn*_)^*T*^ and *A*_*g*_=(*A*_*g*1_,…,*A*_*gn*_)^*T*^ for the vectors of *M* and *A*-values respectively for probe *g*. Note that these vectors are linearly related to *y*_*g*_ through *M*_*g*_=*CMTy*_*g*_ and *A*_*g*_=*CATy*_*g*_ with transformation matrices *CMT*=(−1,1)⊗*I*_*n*_ and *CAT*=(1/2,1/2)⊗*I*_*n*_. Also write *zgT*=(*M*_*g*_, *A*_*g*_)^*T*^ for the combined 2*n*-vector of *M* and *A*-values. Then *z*_*g*_ satisfies the linear model

$$E(z_{g})=Z\beta_{g}$$

where *Z* is the related to the previous design matrix by *Z*=(*C*_*M*_*C*_*A*_)^*T*^*X*.

The linear model for *z*_*g*_ is heteroscedastic, because the first *n* values of *z*_*g*_ have variance $\sigma_{\text{Mg}}^{2}$, whereas the remaining *n* values have variance $\sigma_{\text{Ag}}^{2}$. The model can be fitted using an efficient REML algorithm designed for heteroscedastic regression models [30]. This yields REML estimators ${\hat{\sigma }}_{\text{Mg}}^{2}$ and ${\hat{\sigma }}_{\text{Ag}}^{2}$ from which an estimate of *ρ*_*g*_ can be obtained. This regression approach is more general than the mixed model approach described above in that the estimated intra-spot correlation can take negative as well as positive values. The REML estimators ${\hat{\sigma }}_{\text{Mg}}^{2}$ and ${\hat{\sigma }}_{\text{Ag}}^{2}$ can be shown to follow approximate chisquare distributions with fractional degrees of freedom *d*_*Mg*_ and *d*_*Ag*_ respectively (see Methods).

### Estimating the common intra-spot correlation

The intra-spot correlation *ρ*_*g*_ arises from the technical design of two channel arrays rather than from biological variation or from characteristics of the RNA sources being compared. It is therefore reasonable to assume that the intra-spot correlation will be relatively consistent across the probes. This leads to the argument that the correlation estimators ${\hat{\rho }}_{g}$ may be pooled between probes, an approach similar to that used by [27] when treating within-array replicate probes. From this point, we assume therefore that the intra-spot correlations are equal across probes, *ρ*_*g*_=*ρ* for all *g*.

If the data from different probes were statistically independent, then the REML estimator of *τ*= tanh−1(*ρ*) would be

(2) $$\hat{\tau }=\frac{1}{2}\log\frac{4\sum_{g=1}^{G}{\hat{\sigma }}_{\text{Ag}}^{2}}{\sum_{g=1}^{G}{\hat{\sigma }}_{\text{Mg}}^{2}}.$$

Although theoretically efficient under the independence assumption, this estimator is not robust against outliers.

A more robust estimator can be constructed by obtaining probe-wise estimates of *τ*. The individual probe version of equation (2) is

$${\hat{\tau }}_{g}=\frac{1}{2}\log\frac{4{\hat{\sigma }}_{\text{Ag}}^{2}}{{\hat{\sigma }}_{\text{Mg}}^{2}}.$$

It is shown in Methods that ${\hat{\tau }}_{g}$ is a biased estimator of *τ*, but with a constant bias that can be computed from the degrees of freedom for $\sigma_{\text{Ag}}^{2}$ and $\sigma_{\text{Mg}}^{2}$. The bias correction term is similar to the analogous term derived in [27]. A trimmed mean of the bias-corrected ${\hat{\tau }}_{g}$ across all probes then provides a suitably robust estimator of *τ* and hence of *ρ*= tanh(*τ*). Our software implementation trims 15% of the probes from each tail by default.

### Common correlation inference reduces to ordinary linear modeling

The pooled estimator $\hat{\rho }=\tanh(\hat{\tau })$ is estimated from all the probes on the microarray, typically tens of thousands of probes, and hence can be considered to be a highly precise estimator on which each individual probe has little influence. A consequence of this is that $\hat{\rho }$ can be treated as known when undertaking inference about each individual probe. In particular, the heteroscedastic regression model for *M* and *A*-values described above can be transformed to an ordinary homoscedastic regression model by rescaling all the *M*-values by ${\left\{2(1-\hat{\rho })\right\}}^{1/2}$ and all the *A*-values by ${\left\{(1+\hat{\rho })/2\right\}}^{1/2}$. This leads to a re-scaled version $z_{g}^{*}$ of *z*_*g*_ and a re-scaled version *Z*^∗^ of the design matrix *Z*. The estimator of *β*_*g*_ finally is the ordinary least squares estimator

$${\hat{\beta }}_{g}={\left(Z^{*T}Z^{*}\right)}^{-1}Z^{*T}z_{g}^{*}.$$

In this way, the common correlation model permits us to undertake a separate channel analysis without incurring the inferential complexities of mixed models or heteroscedastic regression. Once the common intra-spot correlation is estimated, the separate channel analysis can utilize the established framework of linear modeling for microarray data [26]. In particular, empirical Bayes methods can be used to borrow strength between probes, leading to moderated *t*-statistics with exact distributions, as previously used for log-ratio analysis or for one-channel microarrays [26]. Details are given in Methods.

### Efficiency gains for separate channels over log-ratios

#### Recovering information from the *A*-values

This section considers the relative efficiency gains of separate-channel versus log-ratio analysis for some commonly used designs. Efficiency is considered on a gene by gene level, so the subscript *g* is suppressed in this section.

Our reformulation of separate channel analysis in terms of *M* and *A*-values clarifies the relationship with log-ratio analysis, because the traditional log-ratio analysis is exactly equivalent to the *M*-value analysis. In other words the log-ratio analysis is equivalent to ignoring the second set of *n* observations in the heteroscedastic regression described above. The extra information recovered in the separate-channel approach compared to analysis of the log-ratios corresponds exactly to the information contained in the *A*-values about the treatment effects of interest. Recovering this information in general improves our power to detect treatment differences.

#### Paired design

The simplest comparative microarray experiment has only 2 treatments and only biological replication. On each array, one channel is hybridized to a sample from each treatment. In this case there is no information gained from recovering intra-probe information. Both the log-ratio and the separate channel approaches yield the same *t*-statistic (the paired t-test) for testing for differential expression for a given probe.

#### Common reference design

The second simplest design also has only 2 treatments (say B and C) and only biological replication. However, one channel on each array is hybridized to a common reference sample R, which is usually a technical replicate from a large RNA pool, while the other channel is used for a sample from one of the 2 treatments of interest. Suppose that there are *n*/2 arrays comparing B with the reference and *n*/2 comparing C with the reference. We will assume that the reference is always labeled green, but the analysis below is readily modified to accommodate dye-swap or dye-balanced designs, in which B and C may be hybridized with either label.

Consider the analysis for a given probe. Let ${\bar{M}}_{B}$ be the mean of the *M*-values for the arrays hybridized with B and ${\bar{M}}_{C}$ be the mean of the *M*-values for the arrays hybridized with C. Define ${Ā}_{B}$ and ${Ā}_{C}$ similarly. Then the laws of probability for sums and differences of random variables give:

$$\begin{array}{lll} {\bar{M}}_{B} & ∼N(\beta_{B}-\beta_{R},4\sigma^{2}(1-\rho )/n)\; & \;\\ {\bar{M}}_{C} & ∼N(\beta_{C}-\beta_{R},4\sigma^{2}(1-\rho )/n)\; & \;\\ {Ā}_{B} & ∼N(\beta_{B}/2+\beta_{R}/2,\sigma^{2}(1+\rho )/n)\; & \;\\ {Ā}_{C} & ∼N(\beta_{C}/2+\beta_{R}/2,\sigma^{2}(1+\rho )/n)\; & \; \end{array}$$

where *β*_*B*_, *β*_*C*_ and *β*_*R*_ are the log-expression values for the probe in RNA samples *B*, *C* and *R* respectively. Write *γ* for the log-ratio of interest, *β*_*B*_−*β*_*C*_. The *M*-values yield the estimator

$${\hat{\gamma }}_{M}={\bar{M}}_{B}-{\bar{M}}_{C}$$

with variance

$$\text{var}{\hat{\gamma }}_{M}=8\sigma^{2}(1-\rho )/n$$

The *A*-values yield the estimator

$${\hat{\gamma }}_{A}=2({Ā}_{B}-{Ā}_{C})$$

with variance

$$\text{var}{\hat{\gamma }}_{A}=8\sigma^{2}(1+\rho )/n$$

The statistical (Fisher) information provided by the *M*-values is $1/\text{var}{\hat{\gamma }}_{M}$ while that from the *A*-values is $1/\text{var}{\hat{\gamma }}_{A}$.Taking the ratio of these two variances shows that the extra information provided by the *A*-values relative to that provided by the *M*-values is

$$\frac{1-\rho }{1+\rho }.$$

If *ρ* is close to one, then the added information is close to zero and the log-ratio analysis is nearly fully efficient. If *ρ* is small, however, then the *A*-values can contribute nearly as much information as the *M*-values, effectively doubling the statistical information that is used.

These formulas also allow us to explore the efficiency of the common reference design itself. Suppose that, instead of hybridizing the reference sample to each array, one channel of each array had been left empty. In that case, the log-fold-change *β*_*B*_−*β*_*C*_ would be estimated using a one channel analysis with variance 4*σ*^2^/*n*. Comparing this with $\text{var}{\hat{\gamma }}_{M}$ shows that the use of competitive hybridizations with a common reference improves the precision of the experiment if and only if the intra-spot correlation is greater than 0.5, assuming that the usual log-ratio analysis is performed. When the intra-spot correlation is less than 0.5, subtracting the reference from each *M*-values increases the variance of the observation rather than decreasing it. On the other hand, when a separate channel analysis is used, the use of the common reference channel always improves the precision of the experiment relative to not observing the reference channel at all. By using all the information available, the separate channel analysis restores the intuition that adding extra observations to a data set should not worsen the results.

#### Unconnected designs

Separate channel analysis is most useful in the case of unconnected designs for which some comparisons cannot be made through the *M*-values. Suppose that *n*/2 arrays are hybridized with RNA from sources B and C and *n*/2 arrays are hybridized with sources D and E. Then the comparisons B versus C and D versus E can be made using *M*-values but B or C vs D or E can not. Let ${\bar{M}}_{\text{BC}}$ be the mean of the *M*-values for the arrays hybridized with B and C and ${\bar{M}}_{\text{DE}}$ be the mean of the *M*-values for the arrays hybridized with D and E. Similarly for ${Ā}_{\text{BC}}$ and ${Ā}_{\text{DE}}$. Then

$$\begin{array}{lll} {\bar{M}}_{\text{BC}} & ∼N(\beta_{B}-\beta_{C},4\sigma^{2}(1-\rho )/n)\; & \;\\ {\bar{M}}_{\text{DE}} & ∼N(\beta_{D}-\beta_{E},4\sigma^{2}(1-\rho )/n)\; & \;\\ {Ā}_{\text{BC}} & ∼N(\beta_{B}/2+\beta_{C}/2,\sigma^{2}(1+\rho )/n)\; & \;\\ {Ā}_{\text{DE}} & ∼N(\beta_{D}/2+\beta_{E}/2,\sigma^{2}(1+\rho )/n)\; & \; \end{array}$$

where *β*_*B*_, *β*_*C*_, *β*_*D*_ and *β*_*E*_ are the log-expression values for the probe in RNA samples *B*, *C*, *D* and *E* respectively. Let *γ*=*β*_*B*_−*β*_*C*_ be the population mean log-ratio between B and C for a given probe and let *δ*=*β*_*B*_−*β*_*D*_ be the population mean log-ratio between B and D for the same probe. The *A*-values provide no information about *γ* so the mixed model approach yields the same estimate and *t*-statistic as the *M*-value linear model approach. This estimator is simply

$$\hat{\gamma }={\bar{M}}_{\text{BC}}$$

with variance

$$\text{var}\hat{\gamma }=4\sigma^{2}(1-\rho )/\mathrm{n.}$$

The *M*-values provided no information about *δ* meaning that the *M*-value approach is unable to estimate *δ*. The combined *M* and *A*-value approach yields the estimator

$$\hat{\gamma }={\bar{M}}_{\text{BC}}/2-{\bar{M}}_{\text{DE}}/2+{Ā}_{\text{BC}}-{Ā}_{\text{DE}}$$

for *δ* with variance

$$\text{var}\hat{\gamma }=4\sigma^{2}/n$$

Notice that the relative efficiency of $\hat{\delta }$ versus $\hat{\gamma }$ is 1−*ρ*. This represents the reduced efficiency of making an indirect contrast between arrays versus a direct contrast between channel hybridized to the same arrays.

As a published example of an unconnected design, consider the experiment of [15]. Jin et al. [15] considered gene expression differences by gender and age (1 and 6 weeks) in two genotypes of Drosophila melanogaster. Each array had a single combination of gender and genotype with both ages in the different channels. Thus this is a paired design in the age main effect, and this is the only effect that can be tested using the log-ratio analysis. Instead a mixed model separate channel analysis was used to analyze the design as a 2×2×2 factorial design.

#### Size of efficiency gains in practice

As noted above, there are two extreme cases in which the efficiency gain from separate channel analysis is either zero or 100%. For the simplest two-color experiment in which two treatments are competitively hybridized on the same arrays, and probe-specific dye-effects are absent, then the log-ratios are fully efficient and there is no information to be gained from a separate channel analysis. The other extreme is that in which unconnected treatments are to be compared. In that case the log-ratios contain no information, and the information gained from separate channel analysis is 100% (or infinity relative to the log-ratio information).

For all other designs, the efficiency gain from separate channel analysis depends on the size of the intra-spot correlation. The intra-spot correlation measures the proportion of total variability arising from technical variability of arrays. Generally speaking, the more biological variability there is relative to technical inter-array variability in an experiment, the lower the intra-spot correlation and the greater the information gain. The titration experiment (see second case study) provides an example of an experiment with technical replication only. In this case, all the variation is technical in nature and the intra-spot correlation is very high at 0.95. The ApoAI knockout experiment (see first case study) is conducted on genetically identical mice. This experiment has a moderate degree of biological variability and yields an intermediate intra-spot correlation of 0.85. The California poppy data (third case study) shows a greater degree of biological variability and yields a relatively low intra-spot correlation of 0.65. For a common reference design, the relative efficiency gain from separate channel analysis with these three intra-spot correlation scenarios would be 2%, 8% and 21% respectively. Note that an efficiency gain of 21% achieves the same improvement in precision as increasing the number of biological samples and arrays by the same percentage.

## Case studies

### Analysis pipeline

This section considers three case studies. The ApoAI knockout data is from academic spotted arrays while the titration example used commercial Agilent arrays. The California poppy study used custom Agilent arrays designed from a shallowly sequenced transcriptome. Unless otherwise noted, all analyses were undertaken using the limma software package [10].

The intensity data were background corrected and normalized prior to differential expression analysis as described in Methods. Briefly, *M*-values were loess normalized [20] using normalizeWithinArrays while *A*-values were quantile normalized [31] using normalizeBetweenArrays. This pipeline normalizes both the *M*-values and the *A*-values in a way that agrees with the usual *M*-value normalization for a traditional log-ratio analysis. The log-ratio and separate channel analyses used the same pre-processed data in each case to ensure the analyses are directly comparable.

Linear modeling used the lmFit function for log-ratio analyses and the intraspotCorrelation and lmscFit functions for separate channel analysis. In all cases the eBayes function was used to construct empirical Bayes moderated *t*-statistics and *p*-values [26].

### ApoAI knockout experiment - a 2 sample common reference design

The apolipoprotein AI gene (known as either Apoa1 or ApoAI) is known to play a pivotal role in high density lipoprotein (HDL) metabolism. Mice which have the ApoAI gene knocked out have very low HDL cholesterol levels. Callow MJ, 2000 [32] discusses an experiment to determine how ApoAI deficiency affects the action of other genes in the liver, with the idea that this will help determine the molecular pathways through which ApoAI operates. A common reference design was used with 16 arrays, 8 wild-type and 8 knock-out mouse liver samples labeled with Cy5 and a common reference sample, created by pooling RNA from the 8 wild-type mice labeled with Cy3. Samples were hybridized to a custom spotted cDNA microarray with 6384 probes.

Both log-ratio and separate channel analyses were conducted to find genes differentially expressed in the ApoAI samples as compared to wild-type. The intra-probe correlation was estimated to be 84.9%, suggesting an efficiency gain of 8% for the separate channel over the log-ratio analysis.

**Figure 1:**
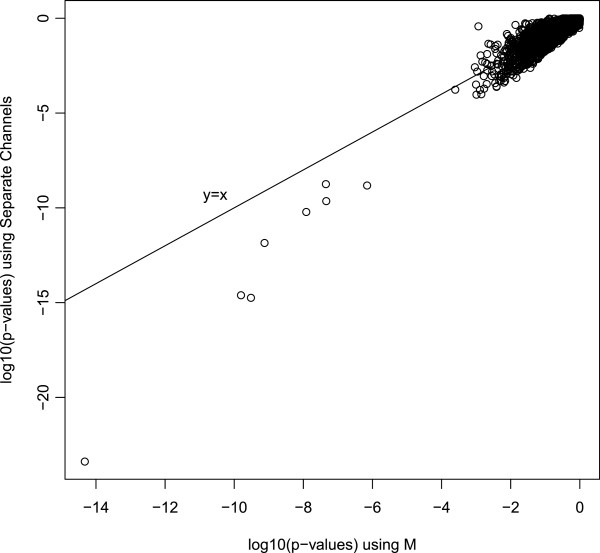
**ApoAI data *log10 *(p-values).** log10(p-values) from the separate channel plotted against the log10(p-values) from the log ratio analysis for the ApoAI data. The p-values for the two methods are correlated, but the p-values from the separate channel analysis are smaller.

Figure 1 relates the *p*-values from the two analyses in a scatterplot on a log10 scale. Both analyses assign very low *p*-values to the same top eight genes, but the *p*-values from the separate channel analysis are several orders of magnitude lower than those from the log-ratio analysis. The separate channel analysis also detects more differentially expressed genes than the log-ratio approach at any false discovery rate (FDR) greater than 0.05 (Table 1).

**Table 1:** Number of significant genes for the ApoAI knockout experiment

**FDR**	**Log-ratio**	**Separate channel**
0.01	8	8
0.05	8	8
0.10	8	15
0.25	9	53

The second and third columns show the number of genes declared differently expressed at various FDR levels by the log-ratio and separate channel analyses respectively. FDRs were estimated using the qvalue package. The separate channel analysis detects more significant genes.

The qvalue package [33] implements Storey’s 2003 method [34] for estimating positive FDRs (*q*-values) and for estimating the total proportion of probes that are not differentially expressed (*π*_0_). The log-ratio and separate channel analyses yielded similar values for ${\hat{\pi }}_{0}$ (86% and 88% respectively). This suggests that there are actually over 700 probes that are truly differentially expressed but were not detected at conventional FDR levels because of small fold changes or high variability. As expected, the separate channel approach shows a gain in power and is able to detect more of these probes. At the same time, the fact that ${\hat{\pi }}_{0}$ does not decrease for the separate channel analysis shows that the separate channel analysis is not on average decreasing the *p*-values of non-differentially expressed genes.

The results in Table 1 are for qvalue FDRs that incorporate the estimate of *π*_0_. The nonadaptive Benjamini and Hochberg FDR estimator [35] gives similar results but is slightly more conservative. If Benjamini and Hochberg FDRs are used, the number of probes detected by the separate channel method decrease slightly to 13 and 44 at FDRs 0.1 and 0.25 respectively.

### Titration data - a multi-treatment common reference design

Two cell lines with very different gene expression profiles were used to prepare a titration series of mRNA as a test of microarray technologies [2]. The two cell lines were MCF7, a cell line derived from breast epithelial cancer cells, and Jurkat, derived from T cell leukemia cells. RNA samples from the two cell lines were mixed in a titration series with 0%, 50%, 76%, 88%, 94%, and 100% MCF7 mRNA. Each mixture was labeled with both red and green and hybridized to 2 arrays. A separate 0% MCF7 sample was used as a reference on each array, in a dye-swap reference design. The data analyzed here were hybridized to Agilent commercial human arrays. The data are available at [36]. After normalization and filtering of very low expressing genes as described in Methods, 17985 spots were used in the analysis.

The analysis was performed as a comparison of each sample with the control sample with 0% MCF7. We expect that the number of genes detected as differentially expressed should increase with the percentage of MCF7 in the sample. Four analysis methods are compared: log-ratio analysis using limma[10] and three separate channel analyses: the ordinary linear mixed model as in [16] computed with lme[37], the common correlation separate channel method implemented in limma and the variance component shrinkage method implemented in maanova[17].

The ordinary mixed model [16] is the classical model for analysis of variance in an incomplete random block design. It includes a block (spot) effect, or equivalently an intra-spot correlation, and an error variance computed for each gene. In the context of microarray data analysis, it has the disadvantage of not borrowing information between probes, so the analysis of each probe relies solely on the data for that probe.

For maanova we used the linear mixed model with array as a random effect, and used the “Fs” option with tabulated p-values to estimate the differential expression p-values. This option imposes shrinkage of the gene-wise mean squared error similar to the method in limma as well as shrinkage of the gene-wise estimate of the random spot variance. However, there are two important differences between maanova and the common correlation separate channel analysis implemented in limma. Firstly, maanova moderates the gene-wise estimate of the spot effect, while the common correlation model imposes a common value *ρ* for the intra-spot correlation for all genes and models the spot variance $\sigma_{\text{gb}}^{2}$ for gene *g* as $\sigma_{\text{gb}}^{2}=\sigma_{\text{ge}}^{2}\rho /(1-\rho )$. Because the common correlation model estimates the common intra-spot correlation *ρ* from the (typically) thousands of spots on the arrays, it is treated as a known constant. Hence the common correlation model adjusts only for the estimation of $\sigma_{\text{ge}}^{2}$ yielding larger error degrees of freedom. As well, because the implementation of the common correlation model in limma uses an empirical Bayes estimator for $\sigma_{\text{ge}}^{2}$, the posterior error degrees of freedom are used for evaluating p-values. maanova uses an empirical method of shrinkage of the estimates of both $\sigma_{\text{gb}}^{2}$ and $\sigma_{\text{ge}}^{2}$ which does not provide a degrees of freedom adjustment. Instead maanova has an option to compute the permutation null distribution of test statistic. However, in this analysis we chose to use the tabulated p-values, which use the usual ANOVA degrees of freedom. These match the degrees of freedom for the tests performed in the mixed model and adjust for estimation of both $\sigma_{\text{gb}}^{2}$ and $\sigma_{\text{ge}}^{2}$.

**Figure 2:**
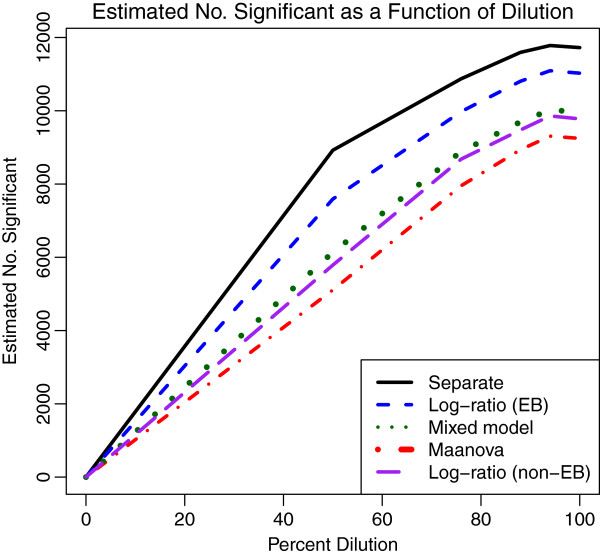
**Number of significant genes for titration data.** The number of significant genes increases with percent MCF7 for all analysis methods (Benjamini and Hochberg FDR <0.01). The separate channel analysis makes the most discoveries at each dilution. The log-ratio method with variance shrinkage is the next most powerful. The linear mixed model which is uses a genewise variance estimator is more powerful than the log-ratio method with genewise variance estimator, but not as powerful as either of the methods using variance regularization. For these data, maanova is the least powerful.

Figure 2 displays the number of genes significant at estimated FDR less than 0.01 using the Benjamini and Hochberg method [35]. The separate channel analysis discovers the most significant genes at all levels of dilution, followed by the log-ratio analysis. The other two analyses have less power. However, all 4 analyses agree on 14774 genes over all levels of dilution above 0. The conclusion is similar if we consider a false discovery rate less than 0.05 for the significance cut-off. While we might expect all the separate channel analyses to outperform the log-ratio analysis, the large intra-spot correlation of 0.95 assures high relative efficiency of the log-ratio analysis with an efficiency loss of less than 2.6%. Hence, the differences in power among the methods is due to both the additional information from recovery of inter-spot information and the variance regularization. To highlight this, the curve for the log-ratio analysis with variance computed genewise without regularization is also plotted. As expected, it lies below the curve for the separate channel analysis using the mixed model.

Of the 8923 genes found significant by the separate channel analysis at 50% dilution and FDR < 0.01, 8354 (94%) are found significant at all higher dilutions and only 134 (1.5%) are found only at this dilution, which corresponds well to the estimated false discovery rate. By contrast, the next most powerful method, the log-ratio method, finds only 7591 significant genes at 50% dilution and FDR < 0.01.

**Figure 3:**
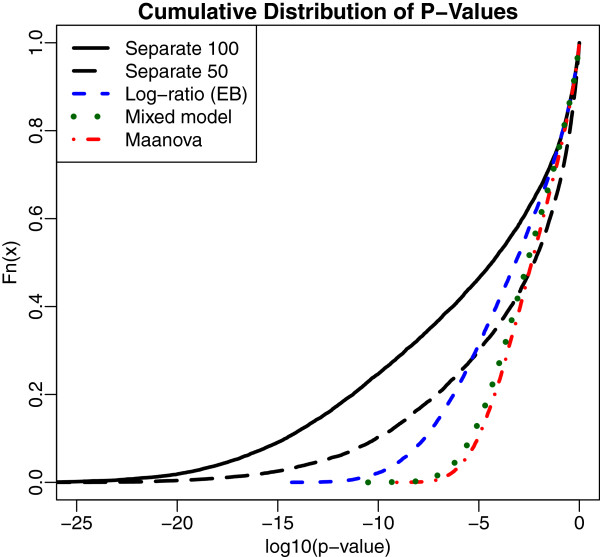
**Cumulative distributions of p-values for the titration data.** The empirical cumulative distribution function of the log10(p-values) for the titration data using four analysis methods. The separate channel p-values are shown in black at 100% and 50% MCF7. The other methods are shown at 100% only. This shows that the separate channel analysis at 50% MCF7 is more powerful than the other methods at 100% MCF7. The log-ratio analysis with variance regularization is more powerful than maanova and the mixed model, but less powerful than the separate channel analysis with variance regularization, although the estimated efficiency gain for the latter is only 2.5%.

To see the comparative results in more detail, Figure 3 shows the empirical cumulative distribution function of the log10 of the p-values for some of the comparisons. Curves farther to the left indicate smaller p-values and hence more powerful tests. For each method, the power of the tests increases as the percentage of MCF7 in the sample increases. In the interests of clarity, we show only a few of the curves. The two black lines are the common correlation separate channel analyses at 50% MCF7 (right-most curve) and 100% MCF7 (left-most curve). The remaining curves are for the 100% MCF7 comparison. The log-ratio analysis, which also uses variance regularization, is the most powerful among the remaining methods, but is only about as powerful as the separate channel analysis at 50% MCF7. The log-ratio analysis without variance regularization yields a curve that is almost indistinguishable from the mixed model. The correlation among the p-values for any pair of methods for the 100% MCF7 treatment is over 93%.

**Figure 4:**
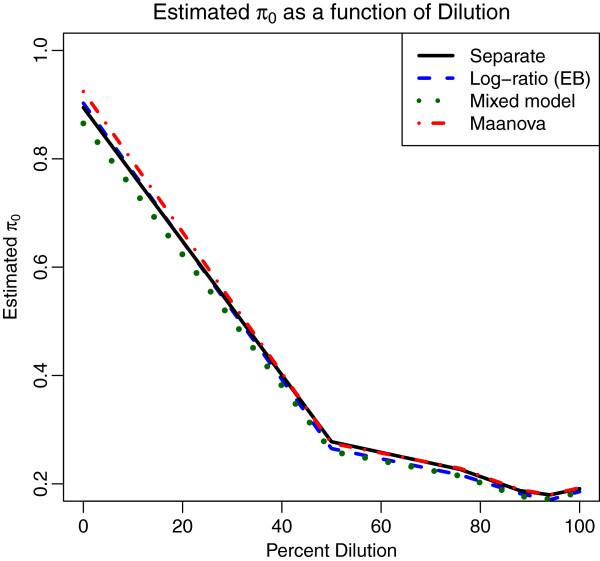
**Estimated*π***_***0***_** for the titration data.** Estimated proportion of non-differentially expressed probes as a function of percent dilution for four different analysis methods. The *x*-axis gives the proportion of MCF7 RNA in the samples at each dilution, the remainder being Jurkat RNA. Each dilution is compared back to pure Jurkat samples. There is very little difference among the estimates for the four methods.

Figure 4 shows the estimated proportion *π*_0_ of nondifferentially expressing genes using the method of Storey [34] as a function of the percentage of MCF7 in the sample for the four analysis methods. Note that the true set of differentially expressing genes does not depend on the percentage dilution (except of course for 0%) so that differences in the estimate of *π*_0_ among the dilution levels is simply due to differences in detection power due to the increasing effect size induced by the dilution. We see that the estimate of *π*_0_ decreases as the percentage of MCF7 in the sample increases, for all analysis methods. The estimates of *π*_0_ at each level of dilution are very similar for all four analysis methods, varying by less than 2% except at the 0% dilution.

**Figure 5:**
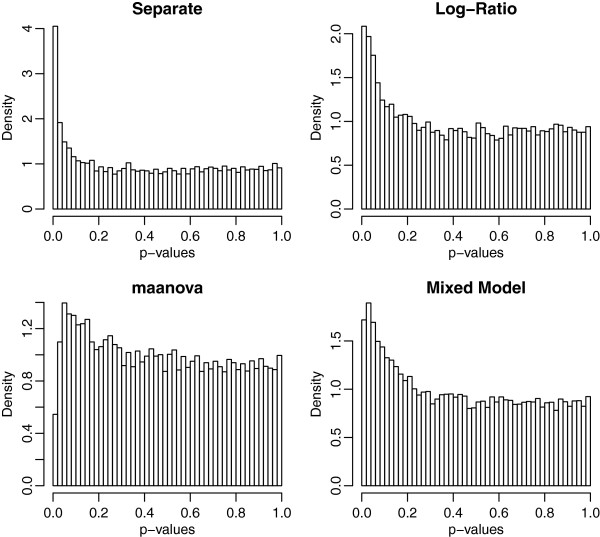
**P-values for the control treatment of the titration data.** For the control treatment (0% MCF7) the 100% of the genes should express nondifferentially. However, for all four analysis methods, the histogram of p-values shows an excess of small p-values indicative of differential expression.

We expect *π*_0_=1 at the 0% dilution, because the comparison is pure Jurkat vs Jurkat, but the estimates actually ranged from 0.87 (for the mixed model) to 0.93 (for maanova). While this cannot be explained on biological grounds, it is possible that some unknown technical effects in handling the arrays introduced some subtle batch effects. Figure 5 displays the unadjusted p-values computed by each of the 4 methods for the 0% MCF7 comparison. The excess of small p-values accounts for the low estimates of *π*_0_. For this comparison, the separate channel method gives 2 significant genes at 1% FDR and 29 at 2% FDR. While this is implausible from the biological point of view, all of the analysis methods indicate that *π*_0_≤0.93 as discussed above, indicating that there may be some unexplained technical effects inducing differential expression among these samples.

### California poppy data - a double loop design

**Figure 6:**
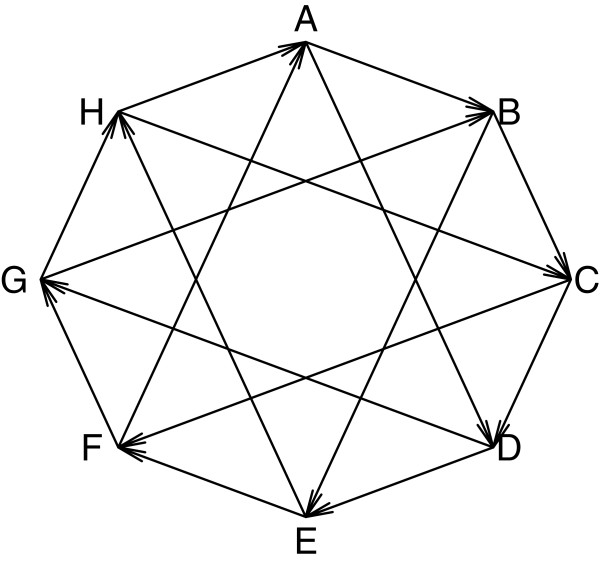
**The double loop design for the poppy experiment.** The double loop design for the poppy experiment. Each letter represents a tissue. Each edge represents a microarray. The head of the arrow represents the red channel and the tail represents the green. Each tissue is represented by 4 samples on 4 arrays.

For an evolutionary study of gene expression in plant organs, tissues from 8 above ground organs were collected from California poppy in 4 biological replicates [38]. The mRNA was labeled so that 2 biological replicates of each tissue were labeled with each dye, and the samples were hybridized to custom Agilent microarrays with 6446 unique poppy probes in a double loop design. The experiment is described in detail in [38] and is illustrated in Figure 6. The design was selected to optimize the pairwise comparisons between certain tissues, while keeping the power of all pairwise comparisons as close as possible with 16 arrays and maintaining balance among the biological replicates.

The estimated intraclass correlation for this experiment is 65.2%. The relative efficiency of the comparisons depends on their relative locations on the double loop in Figure 6[11].

Table 2 displays the number of differentially expressed genes detected for the leaf versus sepal and leaf versus carpel comparisons using log-ratios and separate channel analyses. In Figure 6, leaf tissue is in position A, sepal in position B and carpel in position E. The separate channel analysis detects substantially more significant genes for all comparisons and all FDR cutoffs. Almost all genes detected by the log-ratio approach were also detected by the separate channel method. At FDR <0.01, all but 10 of the 668 genes detected as differentially expressed for leaf versus sepal comparison by the log-ratio analysis were also found by the separate channel analysis. For the leaf vs carpel comparison, all but 1 of the 132 genes found by log-ratio analysis were also found by the separate channel analysis. At the same time, the separate channel analysis does not decrease the estimate of *π*_0_, suggesting that it is not decreasing the *p*-values of non-differentially expressed genes.

**Table 2:** Number of significant genes for the poppy data

			**Number of differentially expressed genes**	
**Analysis**	**Comparison**	${\hat{\pi }}_{0}$	**FDR <0.05**	**FDR <0.01**
Log-ratio	Leaf vs Sepal	0.52	1157	668
Separate channel	Leaf vs Sepal	0.64	1331	954
Log-ratio	Leaf vs Carpel	0.84	254	132
Separate channel	Leaf vs Carpel	0.87	505	314

Rows give results for different tissue comparisons and different analysis methods. The column ${\hat{\pi }}_{0}$ gives the estimated proportion of probes that are not differentially expressed between the tissue types. FDRs and *π*_0_ values were estimated using the qvalue package. The separate channel method detects more significant genes but does not decrease${\hat{\pi }}_{0}$ overall.

## Conclusions

Two-channel microarrays continue to provide cost-effective platforms for whole-genome studies. Technological advances in printing and hybridization have greatly reduced the technical variance associated with microarray studies, while greatly increasing the number of features and reducing cost. Improvements in the statistical analysis of two-channel microarray data further improves cost-effectiveness by improving both sensitivity and specificity especially for small sample sizes.

This study has demonstrated the improvement in efficiency of differential expression analysis that can be achieved for most designs through the use of separate channel analysis. The separate channel analysis can be used to perform comparisons between treatments that may not be possible using log-ratio analysis in unconnected designs. By reparametrizing in terms of spotwise means and differences, the extra information in the separate channel analysis was shown to be that recovered from the *A*-values about the treatment conditions. The insight that the *M* and *A*-values are statistically independent throws some light on why they have been useful quantities for normalization and plotting purposes of two-channel data.

A common correlation estimation strategy has been proposed for the separate channel model, achieving considerable simplification as well as further gains in efficiency by stabilizing the intra-spot correlation estimates. The common intra-spot correlation is more readily interpreted than a set of varying probe-wise estimates, and yields a greater theoretical understanding of how much information is gained from the separate channel analysis. The analysis shows that the efficiency gains are greatest when the intra-spot correlation is small, and this in turn occurs when biological variation is large. In other words, the gain in efficiency is greatest when it is most needed, when biological variation is large.

The common intra-spot correlation strategy is analogous to a common inter-spot correlation strategy proposed previously for combining information across multiple probes for the same gene on the same array [27]]. The common correlation strategy can be viewed as an extreme form of shrinkage estimation in which the prior information about an individual probe *g* is reduced to a point mass at the common value. The current usage applies the same idea to observations from the same spot instead of from different spots in the same array.

Experiments with modest numbers of biological replicates produce variance estimators that are very unreliable on an individual probe-wise basis, so it is important to borrow strength between genes to achieve good statistical power and false discovery control on a genome-wide basis. The common intra-spot correlation approach proposed here facilitates the use of empirical Bayes test statistics with an exact distributional theory in small samples [26]]. This approach cannot usually be applied to mixed models with multiple variance components, because the variance component estimators are not independent and do not follow scaled chisquare distributions. General methods to shrink variance components using James-Stein type estimation have been proposed by Cui *et al*[17]], and these offer an alternative route to regularized separate channel analysis. The resulting test statistics do not however have known null distributions in small samples, leading Cui *et al*[17]] to suggest the use of permutation analysis to establish the null distributions. The common correlation approach of this article provides a more convenient strategy for complex multi-factor experiments.

The separate channel analysis reduces exactly to the log-ratio analysis of [26]] if array is included as a factor in the linear model and the intra-spot correlation is set to zero. This exhibits the fact that the log-ratio analysis uses only within-array information while the separate channel analysis recovers information from between-array variation.

Another possible approach that we have explored is to apply empirical Bayes squeezing to the *M* and *A*-value variances${\hat{\sigma }}_{\text{Mg}}^{2}$ and ${\hat{\sigma }}_{\text{Ag}}^{2}$. Unlike the usual variance components ${\hat{\sigma }}_{\text{eg}}^{2}$ and ${\hat{\sigma }}_{\text{bg}}^{2}$, the M-A variance components are approximately independent and chisquare distributed, and this is exact if the mixed model is balanced, in which case the M-A components are scaled versions of the within and between spot mean squares. Nevertheless the common correlation model, with its aggressive regularization of the intra-spot correlations, appears to be better motivated in terms of technical design of two-channel microarrays.

A number of real data examples with various experimental designs have been used to demonstrate that the common correlation mixed model provides a powerful method for differential expression analysis that outperforms both the log-ratio method and separate channel analyses using the ordinary linear mixed model.

## Methods

### Empirical Bayes moderated *t*-statistics

This section briefly reviews the empirical Bayes differential expression approach implemented in the limma software package. Suppose that probe-wise linear models have been fitted to the expression data as described in Results. Suppose we wish to detect genes for which the *j*th coefficient *β*_*gj*_is non-zero. This coefficient might represent for example a log-fold-change between two treatment conditions. The least squares estimator ${\hat{\beta }}_{g}$ is given in Results. The ordinary *t*-statistic for testing the null hypothesis that *β*_*gj*_ =0 is

$$t_{\text{gj}}=\frac{{\hat{\beta }}_{\text{gj}}}{s_{g}/\sqrt{v_{j}}}$$

where *s*_*g*_ is the residual standard deviation for probe *g* and *v*_*j*_ is the effective sample size for estimating *β*_*gj*_ derived from the design matrix. In the notation used in Results, the effective sample size *v*_*j*_ is the *j*th diagonal element of the inverse of *Z*^∗*T*^*Z*^∗^. Under the null hypothesis, *t*_*gj*_ follows a *t*-distribution on *d*=*n*−*p* degrees of freedom, where *p* is the number of coefficients in the linear model.

Following [25][26]], an improved test can be obtained by computing the posterior variances

(3) $${\tilde{s}}_{g}^{2}=\frac{d_{0}s_{0}^{2}+d\;s_{g}^{2}}{d_{0}+d}$$

and moderated *t*-statistics

$${\tilde{t}}_{\text{gj}}=\frac{{\hat{\beta }}_{\text{gj}}}{{\tilde{s}}_{g}/\sqrt{v_{j}}}$$

Under the null hypothesis,${\tilde{t}}_{\text{gj}}$ follows a *t*-distribution on *d*_0_+*d* degrees of freedom. The gain in degrees of freedom of the moderated over the ordinary *t*-statistic reflects the information which is borrowed from other probes when making inferences about an individual probe. The moderated *t*-test has been shown to be superior to other tests in comparative studies [39][41]].

The hyper-parameters *s*_0_ and *d*_0_ in the prior distribution for $\sigma_{g}^{2}$ are estimated from the expression data on all *G* probes as described by [26]]. After this step, *s*_0_ and *d*_0_ are treated as known.

### Approximate degrees of freedom for the *M* and *A*-value variances

Let *h*=(*h*_1_,…,*h*_2*n*_)^*T*^ be the 2*n*-vector of leverages that arises from fitting the heteroscedastic regression model described in the section on the common correlation model. The leverages are defined to be the diagonal elements of the hat-matrix

$$H_{g}=Z^{*}{\left(Z^{*T}Z^{*}\right)}^{-1}Z^{*T}$$

with *Z*^∗^ defined in Results. The effective degrees of freedom associated with ${\hat{\sigma }}_{\text{Mg}}^{2}$ and ${\hat{\sigma }}_{\text{Ag}}^{2}$ are the residual degrees of freedom associated with the *M*_*gi*_ and the *A*_*gi*_ components respectively of the regression. In terms of the leverages, the effective degrees of freedom are $d_{M}=n-\sum_{i=1}^{n}h_{i}$ and $d_{A}=n-\sum_{i=n+1}^{2n}h_{i}$ respectively. This means that approximately

$$\begin{array}{lll} {\hat{\sigma }}_{\text{Mg}}^{2} & ∼\sigma_{\text{Mg}}^{2}\chi_{d_{M}}^{2}/d_{M}\; & \;\\ {\hat{\sigma }}_{\text{Ag}}^{2} & ∼\sigma_{\text{Ag}}^{2}\chi_{d_{A}}^{2}/d_{A}\; & \; \end{array}$$

The fact that${\hat{\sigma }}_{\text{Ag}}^{2}$ and ${\hat{\sigma }}_{\text{Mg}}^{2}$ follow approximate chisquare distributions implies that ${\hat{\sigma }}_{\text{Ag}}^{2}/{\hat{\sigma }}_{\text{Mg}}^{2}$ follows a scaled *F*-distribution. If we define,

$${\hat{\tau }}_{g}=\frac{1}{2}\log\frac{4{\hat{\sigma }}_{\text{Ag}}^{2}}{{\hat{\sigma }}_{\text{Mg}}^{2}}$$

then it follows that from [27]] that

$$E\left({\hat{\tau }}_{g}\right)=\tanh^{-1}\left(\rho \right)+\text{bias}$$

with

$$\text{bias}=\psi (d_{A}/2)-\psi (d_{M}/2)+\log(d_{A}/2)-\log(d_{M}/2)$$

where *ψ*() is the digamma function. Hence$\left({\hat{\tau }}_{g}-\text{bias}\right)$ is approximately unbiased for *τ*= tanh−1*ρ*.

### Software implementation

The common correlation separate channel approach is implemented in the R [42]] package limma distributed as part of the Bioconductor project [43]]. The intra-spot correlation is estimated by the intraspotCorrelation function, and the separate channel linear model is fitted by the function lmscFit. In other respects, separate channel analyses follow exactly the same framework as other analyses using the limma package. The package includes normalization methods, differential expression analysis and output tabulation for both log-ratio and separate channel analysis.

### Case study datasets

The ApoAI knockout experiment was described by [32]]. RNA samples were hybridized to academic spotted arrays and images were quantified using SPOT. A log-ratio analysis of the microarray data from the experiment is described in Section 8.4.2 of the limma User’s Guide [[44]]. The data were pre-processed for this study as described in the limma User’s Guide except for the addition of quantile normalization of the *A*-values. The microarray data are available as an R data object from the limma package home page [45]].

The titration data have been previously described by [2]]. The original study considered four microarray platforms and three image analysis programs. The current study considers only the Agilent arrays because these were found to be the most precise of the two-color platforms [[2]]. Twenty four RNA samples were hybridized to 12 Agilent Human 1A Oligo microarrays printed with 22,000 oligonucleotide probes. Images were quantified using Agilent Feature Extraction software. Intensities were background corrected by the maximum likelihood normexp method [[28][46]] using the backgroundCorrect function of the limma package. Intensities were global loess normalized using normalizeWithinArrays and *A*-quantile normalized using normalizeBetweenArrays. All positive control probes were filtered before background correction and normalization. After normalization, negative control probes and probes with average log-intensity less than the 75% quantile of the negative controls were filtered from subsequent analysis.

The poppy data have been previously described by [38]] where the probe selection was given in detail. Due to a complex probe design, probes were filtered prior to normalization to select one representative probe per unigene and exclude numerous control probes as described in [[38]]. After filtering, the data were preprocessed by background correction using backgroundCorrect with method=half. Intensities were global loess normalized using normalizeWithinArrays and *A*-quantile normalized using normalizeBetweenArrays.

## Competing interests

The authors declare that they have no competing interests.

## Authors’ contributions

GKS developed and implemented the common correlation analysis. NA did the data analyses. Both authors contributed to the writing of the manuscript. Both authors read and approved the final manuscript.
